# Significance of ST‐Segment elevation in V4R lead in patients with anterior myocardial infarction

**DOI:** 10.1111/anec.12866

**Published:** 2021-06-05

**Authors:** Pooyan Dehghani, Ali Zahedi, Mani Hassanzadeh, Seyed Hosein Alavi, Mansour Jannati, Zahra Mehdipour Namdar, Amir Aslani

**Affiliations:** ^1^ Shiraz University of Medical Sciences Shiraz Iran; ^2^ Cardiovascular Research Center Shiraz University of Medical Sciences Shiraz Iran

**Keywords:** myocardial infarction, ST elevation, V4R lead

## Abstract

**Background:**

There is some evidence of the association between ST‐segment elevation in the V4R chest lead and the likelihood of anterior wall myocardial infarction; however, the link of this phenomenon with the location and the severity of the coronary involvements in such patients remains uncertain. We aimed to investigate the ST‐segment elevation in V4R leads in patients with anterior myocardial infarction and also its effect on prognosis as well as the detection and prediction of the location of arterial stenosis in coronary angiography.

**Methods:**

Data collection was performed by reviewing the hospital recorded files of 195 patients’ suspicion of acute myocardial infarction who have been referred within 2 h of the onset of cardiac symptoms. The patients were then categorized into two groups with and without ST elevation in the V4R chest lead.

**Results:**

Comparing two groups showed a significantly higher rate of concurrent ST‐segment elevation in V_1_ lead in those with ST‐segment elevation in V4R. Echocardiography on the day after anterior myocardial infarction showed LVEF <40% in 74% and 35.2% of patients with and without ST‐segment elevation in V4R, respectively, indicating a significant difference. The lesions on proximal LAD were more common in the group with ST‐segment elevation in V4R.

**Conclusion:**

Our study emphasized a high likelihood of ST‐segment elevation in V4R lead concurrently with ST‐elevation in V_1_ lead. Also, the appearance of ST‐segment elevation in V4R lead can be accompanied with a lower LVEF, myocardial infarct size, involvement of proximal part of LAD, and Wrap around LAD.

## 1 INTRODUCTION

Coronary artery disease is one of the leading causes of death in any community, and electrocardiography (ECG) is one of the fastest and most reliable means of diagnosing myocardial infarction that can provide us with valuable information about the location of significant coronary artery stenosis. Determining the location of coronary artery stenosis in patients with acute myocardial infarction can be clinically helpful in assessing the course of the disease and deciding on the most appropriate treatment (Elsman et al., 2006; Eskola et al., 2009; Yip et al., 2003; Zimetbaum & Josephson, 2003). Anterior wall myocardial infarction is one of the deadliest types of myocardial infarction, which is commonly associated with the left anterior descending artery (LAD) obstruction (Elsman et al., 2006). Many studies have been performed on ECG abnormalities related to the occurrence and prediction of myocardial infarction; however, only little information is available on the association between the chest lead V4R and anterior myocardial infarction (Elsman et al., 2006; Eskola et al., 2009; Yip et al., 2003; Zimetbaum & Josephson, 2003). In general, ST‐segment elevation in the V4R lead can be a sign of right ventricular infarction combined with inferior myocardial infarction (Engelen et al., 1999; Noriega et al., 2014). This condition is also associated with a poorer disease prognosis and occurrence of cardiac arrhythmia (Logeart et al., 2001). Although the right leads are not routinely taken in a twelve‐lead ECG, it must be recorded in the ECG of patients suspected of infarction (Inohara et al., 2013; Ondrus et al., 2013; Zalenski et al., 1998).

In most cases, the inferior and posterior region of the left ventricle, like the posterior region of the right ventricle, receives blood from the right coronary artery, so right ventricular infarction is usually seen in association with inferior myocardial infarction, which also increases the likelihood of in‐hospital mortality and morbidity (Zalenski et al., 1998). In some cases, ST‐segment elevation in the V4R lead can be a sign of anterior myocardial infarction (Elsman et al., 2006; Eskola et al., 2009; Yip et al., 2003; Zimetbaum & Josephson, 2003). In fact, the LAD artery is responsible for the blood supply to the anterior part of the right ventricle. This could explain the occurrence of right ventricular infarction and also the ST‐segment elevation in the V4R lead following the LAD obstruction; however, the location of the LAD artery stenosis is not well defined to justify this event (Walker & Buttrick, 2013). Also, in some cases, despite the appearance of ST‐segment elevation in the V4R lead, the right ventricular infarction does not occur following the LAD artery obstruction, which is called ischemia in neighborhood phenomenon (Barsheshet et al., 2011). We aimed to investigate the ST‐segment elevation in V4R lead in patients with anterior myocardial infarction and also its effect on prognosis as well as the detection and prediction of the location of arterial stenosis in coronary angiography. In this regard, we used parameters to assess the extension of myocardial infarction including (i) the Aldrich scoring formula for estimating myocardial infarction size. This scoring system can effectively determine the myocardial area under potential risk of necrosis, based on ECG scans taken no later than eight hours after the onset of acute myocardial infarction symptoms. This score is calculated using the variables related to ST‐segment elevation, such as the number of leads associated with this change and the sum of ST‐segment elevation amplitudes (considering the J point for such measurement) in some specific leads and (ii) SYNTAX score for scoring the complexity of coronary artery disease based on angiographic grading. In this regard, the SYNTAX score takes into account complex lesions including bifurcations, chronic total occlusions, thrombus, calcification, and small diffuse disease, with the score ranged 0 to greater than 60 in very complex coronary anatomy lesions.

## 2 MATERIALS AND METHODS

### 2.1 Study population

Data collection in this study was performed by reviewing the hospital recorded files of patients who have been referred to the emergency ward at Fatemeh Al‐Zahra Heart Hospital in Shiraz, Iran, within 2 h of the onset of cardiac symptoms and suspicion of acute myocardial infarction. All patients had been triaged for assessing cardiac involvement by ECG followed by conventional coronary angiography, and those with incomplete information were excluded from our study. By reviewing the patients’ files on admission, baseline characteristics including demographics, history of cardiovascular risk profiles (hypertension, hyperlipidemia, diabetes mellitus, obesity, and family history of cardiovascular disorders), and also routine laboratory parameters (complete blood counts, biochemistry, and fasting blood glucose and lipid profiles) were extracted and recorded into the study checklist.

### 2.2 Electrocardiogram

A 14‐lead ECG, which includes 12 cardiac standard leads and two right chest leads, was taken from the patients, and those with the evidence of ST elevation myocardial infarction involving the anterior leads in the ECG were included in the study. The patients were then categorized into two groups with and without ST elevation in the V4R chest lead. We also used the Aldrich scoring formula to calculate and predict myocardial infarction size in patients with anterior myocardial infarction.

### 2.3 Troponin assessment

The first venous blood sample was taken to check cardiac enzyme levels during the first hour of the patient's referral to the hospital. A venous blood sample was retaken 6 h later to check for the changes in the level of enzymes.

### 2.4 Echocardiography

All patients underwent standard cardiac echocardiography within 5 h. Left ventricular ejection fraction (LVEF), mitral regurgitation severity, and wall motion abnormalities were recorded. Three months later, follow‐up echocardiography was performed to evaluate the LVEF.

### 2.5 Treatment

Based on the treatment guidelines, each patient was scheduled for thrombolytic therapy or primary percutaneous coronary intervention. In this regard and using primary or rescue coronary angiography, the stenosis involving at least 50% of the inner surface of coronary arteries sized more than 1.5 mm in diameter was considered significant stenosis. The lesions responsible for the infarct (named as the culprit lesions) were also determined. The following endpoints were finally assessed and compared across the two study groups: (i) Door to Device Time or DTD (defined as the time from arrival to the facility to time of catheterization device deployment in the affected coronary artery); (ii) Infarct‐related artery (referred to a coronary artery that was blocked or stenosed by atheroma and thrombosis, and responsible for acute myocardial infarction); (iii) initial and final TIMI flow; (iv) the location of culprit lesion; (v) the SYNTAX score (to the complexity of coronary artery disease based on angiography findings).

Descriptive analysis was used to describe the data, including mean ± standard deviation (*SD*) for quantitative variables and frequency (percentage) for categorical variables. Chi‐square test, independent *t*‐test, and Mann‐Whitney U test were used for the comparison of variables. For the statistical analysis, the statistical software IBM SPSS Statistics for Windows version 22.0 (IBM Corp. Released 2013, Armonk, New York) was used. *p* values < .05 were considered statistically significant.

## 3 RESULTS

Among the patients who were diagnosed with anterior myocardial infarction, 381 patients were excluded from the present study due to the existing confounding factors. Thus, 195 patients with the definitive diagnosis of the first anterior myocardial infarction were included in the study. Among those, 156 patients had ST‐segment elevation in V4R derivation, and 39 patients did not have such a condition. Also, among the studied patients, 132 patients underwent primary angiography, and 63 patients underwent rescue angiography due to receiving fibrinolytic drugs in another center or to inappropriate response to fibrinolytic drugs. According to the presence or the absence of ST elevation in the V4R chest lead, 156 patients were categorized in the first group and 39 in the latter group. The patients in the two groups were similar in mean age, gender, time to referring the hospital, and baseline cardiovascular risk factors (Table 1).

**TABLE 1:** Baseline information in the two groups

Characteristics	With ST elevation in V4R (*n* = 156)	Without ST elevation in V4R (*n* = 39)	*p*
Mean age, year	58.8 ± 2.23	55.6 ± 2.36	.299
Male gender, %	86 (55.1)	19 (48.7)	.506
History of hypertension	69 (25.0)	16 (41.0)	.718
History of diabetes	43 (27.5)	18 (46.2)	.125
History of smoking	70 (44.9)	22 (56.4)	.197

The most and the least common ECG findings in terms of ST‐segment elevation in the chest leads included extensive anterior and anterolateral walls myocardial infarction and reported in 52.0% and 9.0% of patients, respectively. Comparing two groups with and without ST‐segment elevation in V4R derivation in terms of the changes in anterior chest leads showed a significantly higher rate of concurrent ST‐segment elevation in V_1_ lead in those with ST‐segment elevation in V4R (91.7% vs. 33.3%, *p*‐value = .015) (Table 2). There were significant differences in some baseline cardiac‐related parameters, including LAD, the presence of lesion in proximal LAD, TIMI flow, and the mean Aldrich Score. Abnormal heart rate (as ventricular fibrillation or tachycardia) was observed in 6 of 195 patients (3%) on the first day of hospitalization that all 6 cases occurred in patients with ST‐segment elevation. Echocardiography on the day after anterior myocardial infarction showed LVEF <40% in 74% and 35.2% of patients with and without ST‐segment elevation in V4R, respectively, indicating a significant difference (*p* = .016). Also, by studying echocardiographic data, it was found that 7 out of 195 patients (3%) had mild to moderate mitral valve insufficiency, of which six were associated with ST‐segment elevation in V4R derivation, but without significant difference across the two study groups (*p* = .70). Also, the overall prevalence rate of ventricular septal rupture was 1% occurring in only those with ST‐segment elevation in V4R lead.

**TABLE 2:** Cardiovascular findings in the two groups

Characteristics	With ST elevation in V4R (*n* = 156)	Without ST elevation in V4R (*n* = 39)	*p*
Ventricular arrhythmia	6 (3.8)	0 (0.0)	.213
Type of LAD			
Type I	16 (10.2)	9 (23.1)	.555
Type II	34 (21.8)	13 (33.3)	.060
Type III	106 (67.9)	17 (43.6)	.001
Site of LAD lesion			
Proximal	115 (73.7)	17 (43.6)	.004
Mid part	32 (20.5)	16 (41.0)	.349
Distal	5 (3.2)	3 (7.6)	.670
Other coronary lesions			
LMCA	32 (20.5)	3 (7.6)	.960
RCA	32 (20.5)	13 (33.3)	.467
LCX	12 (7.7)	6 (15.3)	.940
TIMI flow			
0	109 (69.8)	16 (41.0)	.001
1	32 (20.5)	28 (41.0)	.540
2	2 (1.3)	1 (2.6)	.917
3	4 (2.6)	3 (7.6)	.865
Primary SYNTAX score	21.54	19.76	.356
Residual SYNTAX score	2.61	1.32	.591
Aldrich score	14.55	9.98	.001

Also, the LAD lead type III was more prevalent in those with ST‐segment elevation in V4R as compared to those without this evidence (*p*‐value = .013) (Table 3). In addition, the lesions on proximal LAD were more common in the group with ST‐segment elevation in V4R as compared to another group (73.7% vs. 43.5%, *p* = .004). However, the rate of lesions in mid‐portion or distal LAD arteries was not different between the two groups. There was also no difference in the prevalence rate of left main coronary other main coronary artery involvements between the two groups (*p*‐value = .960). In total, of 195 studied patients, 64.1% had single‐vessel, 23.0% had two‐vessel, and 9.2% had three‐vessel involvement. Also, 63 patients (32.3%) received fibrinolytic therapy followed by rescue PCI, while others underwent primary PCI. Among the subjects, 125 patients (64.1%) in angiography had total LAD occlusion with TIMI 0 flow, of which 109 patients were categorized in the group with ST‐segment elevation in V4R and 16 in another group with a significant difference (*p*‐value = .001). The mean SYNTAX score before and after PCI procedure was 21.54 and 2.61 in the group with ST‐segment elevation in V4R and 19.76 and 1.32 in those without ST‐segment elevation in V4R with no significant difference (*p*‐value = .591). Finally, the group with ST‐segment elevation in V4R had a significantly higher mean Aldrich score than those without this change (14.55 vs. 9.98, *p*‐value = .001). Also, the appearance of ST‐segment elevation in V4R lead can also be accompanied with lower LVEF and higher troponin levels (Table 4). Three months later, follow‐up echocardiography revealed lower LVEF in patients with ST‐segment elevation in V4R (Table 4).

**TABLE 3:** Anterior leads involvement in the two groups

Characteristics	With ST elevation in V4R (*n* = 156)	Without ST elevation in V4R (*n* = 39)	*p*
V_1_	143 (91.7)	13 (33.3)	.015
V_1_–V_4_	51 (32.7)	24 (61.5)	
V_1_–V_6_	91 (58.3)	11 (28.2)	
I & AVL	14 (8.9)	4 (10.2)	

**TABLE 4:** Troponin and echocardiographic data in the two groups

	With ST elevation in V4R (*n* = 156)	Without ST elevation in V4R (*n* = 39)	*p*
Troponin on admission (ng/ml)	6.1 ± 0.4	3.2 ± 0.3	.015
Troponin 6 h later (ng/ml)	24.8 ± 2.6	11.5 ± 3.9	.001
LVEF at Hospital (%)	35.2 ± 6.4	47.4 ± 8.6	.016
LVEF 3 months later (%)	36.5 ± 5.7	48.2 ± 8.8	.009
Mild to moderate MR	3 (1.9)	1 (2.6)	.700
Ventricular septal rupture	3 (1.9)	1 (2.6)	.380

## 4 DISCUSSION

Examining the results of the present study showed that the first heart attack was more prevalent in men than women (75.4% vs. 24.6%, respectively). Also, the average age of men with the first heart attack was 56 years, and in women was 60 years. The reason for this discrepancy can be considered in the considerable difference in lifestyle, environmental stress, and the existence of underlying risk factors. A closer look at the present study revealed that the male group was more likely than the female group to have ST‐segment elevation in the V4R lead in association with LAD involvement. Such finding was also associated with more severe coronary involvement, lower LVEF, and also poorer clinical outcome. It was also found that the presence of ST‐segment elevation in V4R was not associated with RCA or LCX involvements or even right ventricular infarction‐related defect. Echocardiography also showed right ventricular systolic failure in none of the patients. As an important result, ST‐segment elevation in V4R lead was significantly related to the site of stenosis in LAD artery (mainly in the proximal zone), also in the Wrap around type where the LAD reaches the heart apex. In other words, ST‐segment elevation in V4R is not limited to RCA involvement. Such results were also revealed in other studies, as shown by Bodi et al. (2010), the appearance of ST‐segment elevation in V4R was associated with the location of LAD involvement.

However, some studies could not demonstrate an association between the presence of ST‐segment elevation in V4R lead and the location of stenosis in coronary artery (Pourafkari et al., 2016). Moreover, similar to our study, other studies found no association between ST‐segment elevation in V4R lead and the baseline cardiovascular risk factors (Bodi et al., 2010; Pourafkari et al., 2016). Also, contrary to our study, Tahirkheli et al. (2000) indicated only 8 out of 88 patients with concurrent anterior and right ventricular infarction. In fact, due to the small number of their patients with RV MI in the study sample, their results may not apply to all patients.

The results of the present study were more consistent with the changes in the ECG as ST‐segment elevation in the anterior thoracic leads, especially in V_1_–V_6_ that was consistent with the study by Engelen et al. (1999). Also, by studying the data, it was found that the majority of patients who had ST‐segment elevation in V4R lead also had ST‐segment elevation in V_1_ lead that might be due to the proximity of the V_1_ derivation axis and the V4R lead. In this regard, it seems that by confirming ST‐segment elevation in V_1_ lead, the likelihood of ST‐segment elevation in V4R can also be expected emphasizing emergent proper treatment procedures because of its association with the risk for lowering left ventricular function and consequent poor prognosis.

In the present study, the angiographic examination of patients did not showed a significant relationship between ST‐segment elevation in V4R lead and involvement of left main coronary artery (LMCA), right coronary artery (RCA), and left circulatory artery (LCX). Also, through assessing the patients’ ECG and echocardiographic data, we did not find a significant relationship between ST‐segment elevation in V4R and rupture of the ventricular septum (VSR) and also mitral valve insufficiency as well as ventricular arrhythmia. However, because all patients with such events had all the evidence of ST‐segment elevation in V4R, appearing the change in V4R lead may be a powerful predictor for such cardiac phenomena.

## 5 CONCLUSION

Our study emphasized a high likelihood of ST‐segment elevation in V4R lead concurrently with ST‐segment elevation in V_1_ lead. Also, the appearance of ST‐segment elevation in V4R lead can also be accompanied with lower LVEF, myocardial infarct size, involving the proximal site of LAD, and also Wrap around LAD and thus detecting ST‐segment elevation in V4R lead can help to predict poorer clinical outcome in affected patients.

## CONFLICT OF INTEREST

The authors have stated explicitly that there are no conflicts of interest in connection with this article.

## AUTHORS’ CONTRIBUTION

All authors reviewed and approved the manuscript. P. Dehghani and M. Hassanzadeh directed this study. S.H. Alavi, Z. Mehdipour Namdar, A. Zahedi, A. Aslani, and P. Dehghani collected patient data. A. Aslani, M. Hassanzadeh, and M. Jannati performed statistical analysis. M. Jannati and Z. Mehdipour Namdar wrote the main manuscript.

## ETHICAL APPROVAL

The current study was carried out in accordance with the declaration of Helsinki and was approved by Shiraz University of Medical Sciences ethics committee.

## Data Availability

The data that support the findings of this study are available from the corresponding author upon reasonable request.
